# Adsorption of Nitrogen Dioxide on Nitrogen-Enriched Activated Carbons

**DOI:** 10.3390/ijms25084421

**Published:** 2024-04-17

**Authors:** Aleksandra Bazan-Wozniak, Agnieszka Nosal-Wiercińska, Judyta Cielecka-Piontek, Selehattin Yilmaz, Robert Pietrzak

**Affiliations:** 1 Department of Applied Chemistry, Faculty of Chemistry, Adam Mickiewicz University in Poznań, Uniwersytetu Poznańskiego 8, 61-614 Poznań, Poland; aleksandra.bazan@amu.edu.pl; 2 Department of Analytical Chemistry, Institute of Chemical Sciences, Faculty of Chemistry, Maria Curie-Sklodowska University in Lublin, Maria Curie-Sklodowska 3, 20-031 Lublin, Poland; agnieszka.nosal-wiercinska@mail.umcs.pl; 3 Department of Pharmacognosy, Faculty of Pharmacy, Poznan University of Medical Sciences, Rokietnicka 3, 60-806 Poznań, Poland; jpiontek@ump.edu.pl; 4 Department of Chemistry-Analytical Chemistry, Faculty of Science, Canakkale Onsekiz Mart University, 17100 Canakkale, Turkey; seletyilmaz@hotmail.com

**Keywords:** activated carbon, chemical activation, ammoxidation, physiochemical properties, NO_2_ adsorption

## Abstract

The aim of this study was to obtain nitrogen-enriched activated carbons from orthocoking coal. The initial material was subjected to a demineralisation process. The demineralised precursor was pyrolysed at 500 °C and then activated with sodium hydroxide at 800 °C. Activated carbon adsorbents were subjected to the process of ammoxidation using a mixture of ammonia and air at two different temperature variants (300 and 350 °C). Nitrogen introduction was carried out on stages of demineralised precursor, pyrolysis product, and oxidising activator. The elemental composition, acid-base properties, and textural parameters of the obtained carbon adsorbents were determined. The activated carbons were investigated for their ability to remove nitrogen dioxide. The results demonstrated that the ammoxidation process incorporates new nitrogen-based functional groups into the activated carbon structure. Simultaneously, the ammoxidation process modified the acid-base characteristics of the surface and negatively affected the textural parameters of the resulting adsorbents. Furthermore, the study showed that all of the obtained carbon adsorbents exhibited a distinct microporous texture. Adsorption tests were carried out against NO_2_ and showed that the carbon adsorbents obtained were highly effective in removing this gaseous pollutant. The best sorption capacity towards NO_2_ was 23.5 mg/g under dry conditions and 75.0 mg/g under wet conditions.

## 1. Introduction

The precursors of activated carbons are waste materials such as fruit stones [1], nut shells [2], or used waste tyres [3]. Activated carbons are also produced from lignin [4], peat [5], wood [6], or cellulose [7]. The precursors of activated carbons can also be fossil carbons [8], which have their own original microporous structure, but these pores are too small and, therefore, are inaccessible to most adsorbents. In order to use them in industry and increase their adsorption properties, they need to be subjected to additional treatment [8].

Activated carbons derive their physicochemical properties not only from their well-developed specific surface area, but also from their heteroatoms. These heteroatoms can either replace carbon atoms within the graphene layer structure or exist as functional groups at the edges of these layers [9]. Heteroatoms exhibit significant diversity in chemical reactivity, as well as variations in their distribution. They are found in various positions, encompassing oxygen, nitrogen, sulphur, phosphorus, halogen atoms, and other elements [10]. The modification of both the precursors and the activated carbons themselves can influence the physicochemical properties of the resulting product. The chemical structure of the activated carbon surface significantly affects its adsorption, electrochemical, catalytic, oxidation-reducing, acid-base, and other properties [11]. Activated carbons featuring a basic surface character hold significant value as materials. Nitrogen can be introduced to create basic sites on the activated carbon surface. Ammonia, urea, and amines are among the most commonly employed reagents for incorporating nitrogen into the carbon structure [12].

A highly efficient technique for enriching carbonaceous materials with nitrogen is through ammoxidation [13]. This process involves treating carbon material with a mixture of ammonia and air at elevated temperatures. A concurrent process of oxidation and nitriding takes place during ammoxidation, resulting in substantial alterations to the material’s chemical structure. The ammoxidation process serves as a means to incorporate considerable nitrogen content into the carbon structure, resulting in a material characterised by a more well-developed total surface area and increased microporosity compared to nitrogen-unmodified coals [14].

Nitrogen dioxide (NO_2_) is an inorganic air pollutant that has harmful effects on human health, animal life, and the environment. It is released as NOx gas due to natural phenomena such as volcanic eruptions, lightning strikes, and forest fires. Recently, nanostructured metal oxides like ZnO, TiO_2_, and CuO have been widely used as components for gas sensors because of their unique structure and surface-to-volume ratio compared to layered materials [15,16,17,18,19,20,21]. On the other hand, composites containing MOFs in their systems are also used for NO_2_ adsorption [22,23].

The primary objective of this research was to produce nitrogen-enriched activated carbons from orthocoking coal by reacting it with gaseous ammonia and to evaluate their physicochemical and sorption properties. The ammonia–carbonaceous material reaction was conducted under ambient air conditions. Nitrogen was introduced during every phase of the activated carbon preparation, including the precursor (demineralised) stage, pyrolysis process, and activation stage. Activated carbons obtained by chemical activation with sodium hydroxide were subjected to a suitability assessment for the removal of iodine and nitrogen(IV) oxide. The tested adsorbents were subjected to detailed elemental and technical analysis and their acid-base properties, as well as textural parameters, were determined.

## 2. Results and Discussion

### 2.1. Characterisation of the Starting Material

The research was conducted on orthocoking coal from the Jas-Mos coal mine (Poland). The main part of the research was carried out on demineralised coal. The technical (Table 1) and elemental analysis of the starting (Precursor) and demineralised (Precursor_dem_) material (Table 2) was the prelude to the study. The analysis of the data obtained shows that treatment with concentrated hydrochloric acid and then hydrofluoric acid resulted in a significant removal of mineral matter and a slight decrease in volatile compounds. Furthermore, the removal of minerals from the precursor did not noticeably impact the individual element content in comparison to the resulting demineralised carbon. The Precursor sample had a higher elemental carbon content by 2.2 wt. % in comparison to the initial material. In contrast, there was a slight reduction in the other elements as determined in comparison to sample J. This decrease was within the range of 0.1 to 2.4 wt. %.

The first method for producing nitrogen-enriched activated carbons involved reacting Precursor_dem_ carbon with ammonia under oxidising conditions, followed by pyrolysis and activation. As shown in the data presented in Table 2, the reaction of demineralised carbon with ammonia under oxidising conditions resulted in a significant reduction in the carbon and hydrogen content. This reduction was more pronounced for the Precursor_demN1_ adsorbent. In contrast, there was a notable increase in nitrogen content, attributed to the incorporation of nitrogen into the carbon structure. The Precursor_demN_ and Precursor_demN1_ carbons exhibited 5.1% and 7.2% higher N^daf^ content, respectively, compared to the JD sample. The higher temperature of the ammoxidation process is favourable for incorporating more nitrogen into the carbon structure. Additionally, the oxygen content increases as a result of oxidation during the ammoxidation process. The pyrolysis of the nitrogen-enriched demineralised coals Precursor_demN_ and Precursor_demN1_ resulted in increased elemental carbon content and decreased hydrogen, nitrogen, and oxygen contents, while the amount of sulphur remained unaffected. The pyrolysis products obtained (Precursor_demN_P, Precursor_demN1_P) were also found to possess a slightly elevated mineral content in comparison to the Precursor_dem_, Precursor_demN_, and Precursor_demN1_ samples. Subsequent detailed analysis of the data enabled us to deduce that the activation process utilising sodium hydroxide for the nitrogen-enriched pyrolysis products resulted in an augmentation of the elemental carbon content within the resultant activated carbons. Simultaneously, this activation process led to a reduction in hydrogen, sulphur, and nitrogen content. The significant reduction in nitrogen content may be attributed to the low resistance of the nitrogen groups to the high activation temperature and agent. Additionally, the activation process resulted in a reduction in oxygen content. The obtained NPA and N1PA carbons had a small amount of ash present in their structures, accounting for 1.0 wt. % and 1.1 wt. %, respectively.

The second method of producing nitrogen-enriched activated carbons involved obtaining the pyrolysis product from demineralised carbon, followed by a reaction with ammonia, and concluding with an activation process (Table 2). As evident from the acquired data, pyrolysing the Precursor_dem_ sample at 500 °C did not yield substantial alterations in carbon and sulphur content when contrasted with the original coal. In contrast, the process resulted in a decrease in hydrogen and nitrogen content and an increase in oxygen content. The reaction of the P sample with ammonia under oxidising conditions resulted in a decrease in carbon and hydrogen content and an increase in oxygen content. Nonetheless, it is noteworthy that these changes were greater when the ammoxidation process was carried out at 350 °C. The ammoxidation process enabled higher nitrogen amounts to be introduced into the PN and PN1 samples than the P adsorbent. The activation of nitrogen-enriched PN and PN1 samples resulted in an increased elemental carbon content and a decreased hydrogen, nitrogen, and sulphur content. The decrease in nitrogen content resulted from the activating agent and the low thermal stability of the nitrogen groupings introduced during pyrolysis. The activation of ammoxidised samples led to a reduction in oxygen content in the resulting activated carbons. It should be noted that both the pyrolysis product and the resulting ammoxidised coals and activated carbon were contained a low mineral content, ranging from 0.7 to 0.9 wt. %.

The third method of producing nitrogen-enriched activated carbons involved subjecting the SPD sample to an activation process, followed by a reaction with ammonia.

The activation process led to an increase in elemental carbon content. A significant decrease in hydrogen and nitrogen content and the complete removal of sulphur was also observed. It can be seen that the PA sample was had minimal nitrogen content, which was related to the low resistance of the nitrogen groups contained in the P sample to the activation agent used in the study and the high temperature of the activation process.

The activated carbons subjected to ammonia treatment under oxidising conditions exhibited a decrease in C^daf^ and H^daf^ content in comparison to the initial activated carbon (PA). Furthermore, it was noted that ammoxidised carbon at 350 °C exhibited a higher nitrogen content compared to the nitrogen-enriched activated carbons produced with ammonia.

Summarising the results presented in Table 2, it is evident that all activated carbons, irrespective of the method of nitrogen enrichment, had a low sulphur content. This is advantageous from an environmental protection standpoint. Furthermore, the activation process performed at different stages of carbon adsorbent production led to an increase in the carbon content (C^daf^) due to the carbon structure’s aromatisation. At the same time, it decreased the hydrogen (H^daf^) and oxygen (O^daf^) content. The data in Table 2 show that activated carbons subjected to the ammoxidation process in the last stage had the highest content of nitrogen (N^daf^). By analysing the data presented in Table 2, it was deduced that the optimal approach for producing nitrogen-enriched activated carbons involves pyrolysis of the demineralised carbon, activating the resulting carbon, and, subsequently, reacting it with ammonia.

**Figure 1:**
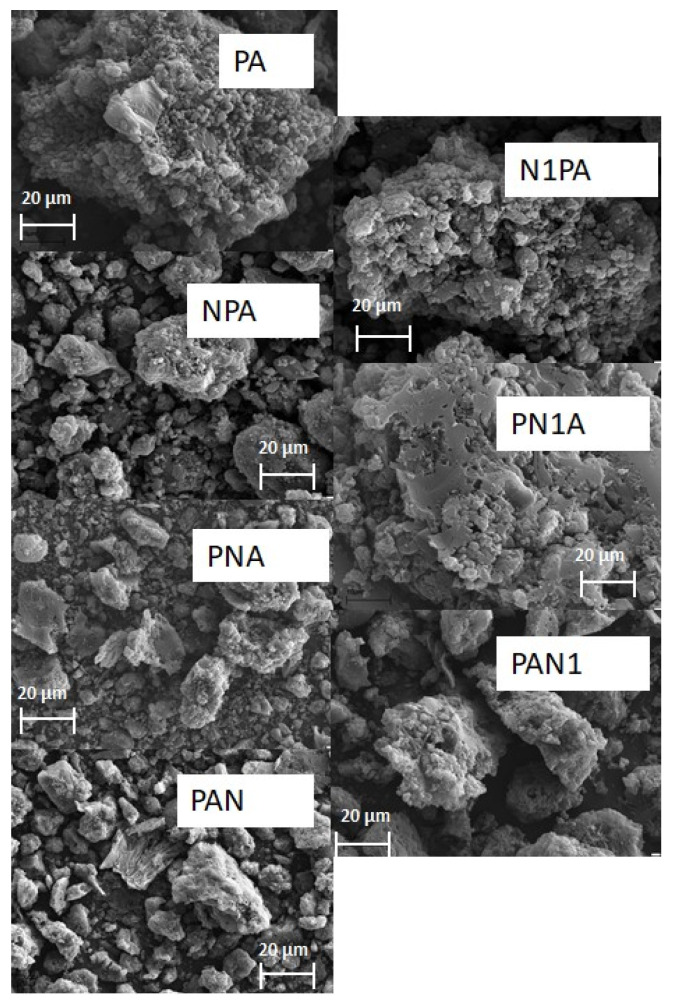
SEM images of activated carbons.

**Figure 2:**
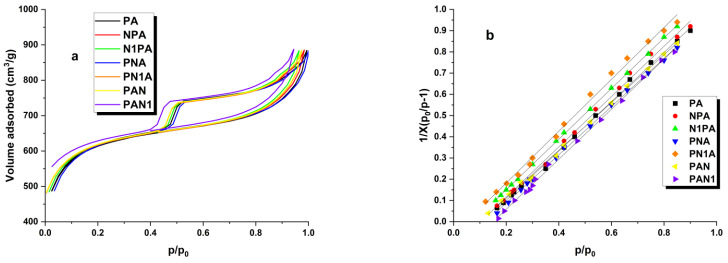
Low-temperature N_2_ adsorption–desorption isotherms (**a**) and fitted plot of BET adsorption isotherm for nitrogen on activated carbons (**b**).

Specific surface area measurements were conducted for all activated carbons produced through precursor pyrolysis at 500 °C, followed by activation with NaOH at 800 °C. The outcomes of these experiments are presented in Table 3. Additionally, SEM images are presented in Figure 1 and the low-temperature nitrogen adsorption/desorption isotherm is presented in Figure 2. The shape of the recorded isotherms is similar to type IV(a) according to the IUPAC classification. Various parameters were assessed, including specific surface area, micropore area, total pore volume, micropore volume, mesopore volume, volume of pores less than 1 nm, and average pore diameter. From the data provided in Table 3, it is evident that the activated carbon derived from PA exhibited an impressive specific surface area, primarily composed of micropores. This carbon material showcased an impressive surface area of 2027 m^2^/g, with micropores accounting for nearly 97% of all of the pores within its structure. Furthermore, the microporous nature of the PA sample was affirmed by the average pore diameter of 2.19 nm. Further analysis of the results indicated that the structural characteristics of nitrogen-enriched activated carbons remained unaltered by the introduction of nitrogen to the carbon material. However, upon comparing the obtained outcomes, it is apparent that the surface area of the samples enriched with nitrogen during the carbon demineralisation phase surpassed that of coals enriched with nitrogen during pyrolysis, and was comparable to those enriched with nitrogen during the activated carbon stage. Additionally, the temperature at which nitrogen was introduced exerted a slight influence on the surface parameters. When a lower temperature was employed, the surface area of the resultant carbons only marginally surpassed that of the modified carbons synthesised at a higher temperature.

In order to characterise the surface properties of the activated carbons produced, we determined the content of the surface oxygen functional groups, both acidic and basic, according to the Boehm method (refer to Table 4). The activated carbons produced by the pyrolysis of demineralised orthocoking coal, followed by NaOH activation, had an acidic surface character. The PA sample contained 1.03 mmol/g of acid groups and 0.71 mmol/g of basic groups in its structure. From the data presented in Table 4, it is evident that the nitrogen-enriched activated carbons contain basic and acidic oxygen functional groups on their surface, with a predominance of basic groups. The results also indicate that, in the case of nitrogen incorporation, basic groups dominate acid groups for the P sample after sodium hydroxide activation.

The measurements showed that unmodified activated carbon contains a significantly higher level of acidic oxygen groups compared to the basic ones. Regarding activated carbons enriched in nitrogen with ammonia, the group content is predominantly dependent on the stage of carbon reaction with ammonia. Activated carbons enriched with nitrogen, following the process sequence of demineralisation → pyrolysis → activation → ammonia reaction, exhibited the most pronounced predominance of basic functional groups compared to acidic groups. Conversely, activated carbons obtained through the sequence of demineralisation → ammonia reaction → pyrolysis → activation displayed the lowest prevalence of basic groups over acidic ones.

### 2.2. Adsorption Study Iodine Number/NO_2_

Table 5 displays the outcomes of iodine adsorption for the investigated activated charcoals. The non-ammoxidation carbon was found to be the most effective adsorbent. The PA sample possessed a sorption capacity for iodine as high as 2045 mg/g. On the other hand, the 300 °C ammoxidation carbon at the stage of demineralised precursor was determined to be the least effective adsorbent. The iodine removal efficiency for the N1PA sample was measured at 1699 mg/g. Similar to the specific surface area, the ammoxidation process resulted in a decreased sorption capacity towards iodine for the nitrogen-enriched activated carbons. The iodine adsorption values for the ammoxidised coals ranged from 1699 to 2029 mg/g. However, the nature of these changes varied among the samples. The lowest iodine adsorption capacity was exhibited by the carbons that underwent nitrogen enrichment during the demineralisation stage. In contrast, the highest iodine sorption capacities were displayed by coals in which nitrogen groups were incorporated into their structure during the activation stage. The data also indicated that slightly improved sorption capacities for iodine were demonstrated by adsorbents that were subjected to ammoxidation at a higher temperature, specifically 350 °C.

The development of the porous structure in activated carbons and their capability to eliminate impurities of sizes similar to 1 nm is indicated by the iodine sorption capacity. A summary of the iodine numbers for carbon adsorbents, which are produced by chemically activating different precursors using activators like H_3_PO_4_, H_2_SO_4_, KOH, and NaOH, is provided in Table 5. Regarding the activator orthophosphoric(V) acid, the iodine numbers, as presented in Table 5, were notably lower than the outcomes achieved for the JDPA sample and the nitrogen-enriched coals. These figures ranged from 576 to 1628.95 mg/g. However, it should be pointed out that in the study conducted by [24,25,26], waste materials were used as precursors for the carbon adsorbents. In the study by [24], rice husks were combined with a 1 M H_3_PO_4_ solution and subsequently carbonised at 400 °C in a muffle furnace for 60 min. The resultant activated carbon was further altered to create a ZnO-containing nanocomposite, and its iodine sorption capacity was measured at 660.4 mg/g. The sorption capacity of the activated carbon (1628.95 mg/g), as outlined in [25], was similar to the outcome achieved for the N1PA sample. This carbon material was generated through a two-step process: initially, sawdust was subjected to carbonisation in a muffle furnace at 600 °C for 1 h, followed by mixing the carbonised product with a 1 M H_3_PO_4_ solution and subsequent activation at 600 °C for 75 min in the second step. Table 5 also displays the iodine numbers of adsorbents obtained through activation using H_2_SO_4_ solution [27], as well as a mixture of H_3_PO_4_ and H_2_SO_4_ [28]. The iodine number of the adsorbent derived from the activation of immature G. hirsutum seeds with a 98% concentrated H_2_SO_4_ solution at a biomass/activator weight ratio of 1:4 was 510 mg/g. This capacity was notably lower compared to the outcomes achieved for samples derived from orthocoking carbon. In the second study, Peltophorum pterocarpum leaves were impregnated with a mixture of sulphuric acid (40 wt. %) and phosphoric acid (85 wt. %), followed by carbonisation in a muffle furnace for 120 min at 550 °C. The resultant activated carbon exhibited an iodine removal efficiency of 1508.76 mg/g. Consequently, it can be concluded that the biomass activation described in works [27,28] also led to adsorbents with decreased sorption capacities towards iodine in comparison to the carbons detailed in this paper. However, it is important to note that the cost of synthesising the adsorbents mentioned in papers [24,25,26] as well as in papers [27,28] is potentially lower when contrasted with activated carbons obtained from orthocoking carbon. Hydroxides, specifically potassium or sodium hydroxide, is a commonly used activator in the synthesis of activated carbons. Hence, sorption capacities comparable to the outcomes presented in this article were observed in earlier studies [29,30]. For instance, activated carbon originating from baobab seed hulls [29] exhibited an iodine adsorption capacity of up to 1854.2 mg/g after impregnation with potassium hydroxide at a mass ratio of 0.3, followed by activation at 800 °C for 2 h under a nitrogen atmosphere. This result significantly surpassed the iodine adsorption values obtained for nitrogen-enriched coals during the demineralised precursor stage, and slightly lagged behind the samples obtained through the demineralisation → pyrolysis → reaction with ammonia → activation procedure. Comparable or even higher sorption capacities were demonstrated by nitrogen-enriched coals derived from a highly volatile bituminous coal in Poland’s Kazimierz-Juliusz mine [30]. These adsorbents were produced using the same method as coals sourced from orthocoking coal from the Jas-Mos mine, but employing potassium hydroxide as the activator.

Table 5 provides a summary of the iodine numbers for activated carbons produced using sodium hydroxide [31,32]. The sorption capacities are reported as 1062.8 mg/g for the carbon derived from palm kernel shells and 913 mg/g for the carbon obtained from pine cones, respectively. The carbon material described in paper [31] was synthesised through the chemical mixing of the initial material with a 0.1 M NaOH solution, without undergoing any thermochemical treatment. On the other hand, paper [32] details a two-stage process for the creation of a carbon adsorbent. Firstly, pine cones undergo pyrolysis in a horizontal furnace at 400 °C. Subsequently, the resulting product is impregnated with a refined activating agent in a weight ratio of 2:1 (NaOH/precursor) before being activated at 600 °C for 20 min.

**Figure 3:**
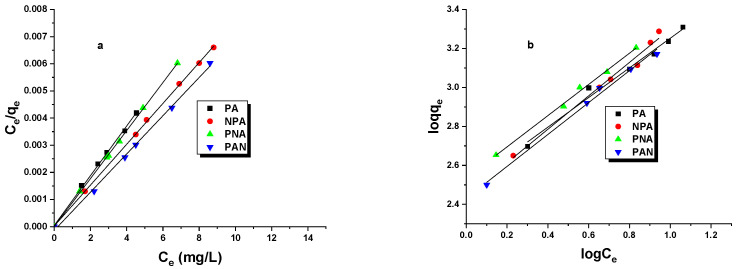
Iodine isotherms fitted to Langmuir model (**a**) and Freundlich model (**b**).

In Table 6, constants for the Langmuir and Freundlich models were calculated for the PA, NPA, PNA, and PAN carbons. The choice of a model that more accurately describes the interactions between the adsorbate and the adsorbent is based on the determination coefficient, R^2^. As indicated by the data in Table 6, all iodine adsorption isotherms can be described by the Langmuir model, suggesting the formation of a monolayer of adsorbate on the adsorbent surface. Furthermore, a higher value of the constant K_L_ implies greater selectivity of the activated carbon for the adsorbate. The highest value was obtained for the PAN sample. In the Freundlich equation, the constant 1/n represents the homogeneity coefficient dependent on the affinity of the adsorbate to the adsorbent. The closer to zero the constant 1/n is, the greater the affinity. Based on the data in Table 6, it can be observed that PNA carbon exhibits the highest affinity for the adsorbate. The course of the isotherms shown in Figure 3 additionally confirms the formation of an adsorption monolayer on the surface of the activated carbons investigated.

All of the obtained activated carbons were subjected to adsorption testing against NO_2_ under both dry and wet conditions, and the results are presented in Table 7. The highest sorption capacity towards NO_2_ was exhibited by the PAN1 carbon, with a capacity of 23.5 mg/g under dry conditions and 75.0 mg/g during tests conducted with steam. It is worth noting that the sorption capacity of the PAN1 sample was higher than that of the PAN1 sample, which underwent oxidation at 300 °C. Specifically, the sorption capacities were 23.0 mg/g and 73.3 mg/g for dry and wet conditions, respectively. In contrast, the unmodified PA activated carbon demonstrated sorption capacities of 22.8 mg/g and 69.3 mg/g for dry and wet conditions, respectively, against the tested contaminant.

In the case of activated carbons that were subjected to ammonia reaction at the demineralised carbon stage, it is observable that the samples thus obtained display the lowest sorption capacity towards nitric oxide(IV) among all the tested adsorbents. The results obtained are more than two times lower under dry conditions and more than four times lower under wet conditions compared to the capacities observed for PAN and PAN1 carbon. On the other hand, a comparison of the results of NO_2_ sorption capacities for activated carbons enriched with nitrogen at the pyrolysis product stage indicates that they were slightly higher in relation to carbon adsorbents obtained according to the demineralisation → ammonia reaction → pyrolysis → activation scheme. However, these capacities were notably lower in comparison to PAN, PAN1 carbons, and unmodified PA carbon. The data presented in Table 7 also demonstrate that samples subjected to ammoxidation at higher temperatures exhibited an improved sorption capacity towards NO_2_ when compared to samples ammoxidised at 300 °C.

The sorption capacities of the obtained samples were notably influenced by the conditions under which the process took place. The sorption capacities are summarised in Table 7, illustrating that all activated carbons exhibited significantly higher sorption capacities during adsorption in the presence of water vapour. This parameter had the most pronounced impact on the ammoxidised carbons at the activation stage and the unmodified JDPA carbon. In contrast, the NPA and N1PA samples were found to be the least influenced by this parameter.

The nitrogen content of the sample structure can be used to determine the sorption capacities of the tested activated carbons. According to the data presented in Table 2, adsorbents PAN and PAN1 exhibited the highest nitrogen removal efficiency and contained the highest amount of N^daf^. On the other hand, NPA and N1PA samples, which had the least amount of nitrogen in their structure, showed the lowest removal efficiency of the test gas. The results shown in Table 7 are also influenced by the specific surface area and porous structure. The activated carbons’ microporous nature promotes NO_2_ adsorption (Table 3). The chemical composition of the samples appears to affect the amount of NO_2_ adsorbed on the activated carbons produced. Upon analysis of Table 4 and Table 7, a trend is observed where samples with a higher concentration of basic groups on the surface of the adsorbents exhibit a higher sorption capacity. Reactions are likely to occur between the functional groups of activated carbons and the adsorbed gas. Furthermore, the mineral substance present in the structure of the obtained samples may also affect the results presented in Table 7. This effect is particularly visible for PAN and PAN1 coals.

**Figure 4:**
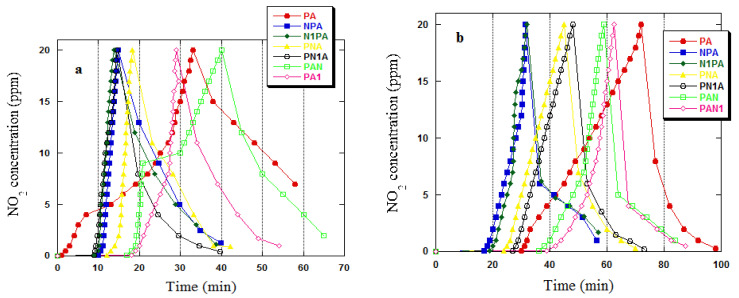
NO_2_ breakthrough curves in dry (**a**) and wet (**b**) conditions for activated carbons.

**Figure 5:**
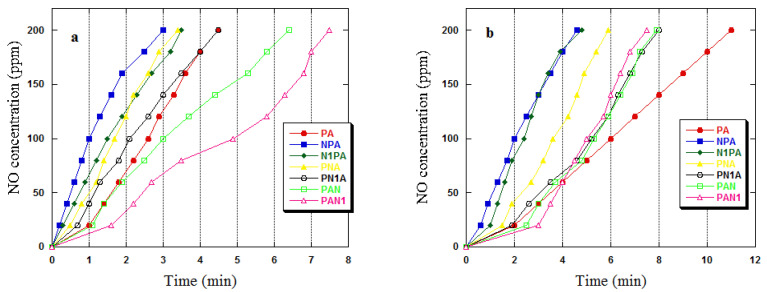
NO concentration curves in dry (**a**) and wet (**b**) conditions for activated carbons.

The NO_2_ breakthrough curves for all samples are displayed in Figure 4. Additionally, Figure 5 presents the NO concentration curves, given that nitric(II) oxide is generated by NO_2_ reduction on the surface of carbon adsorbents. Moreover, activated carbons act as catalysts for NO_2_ decomposition into NO [33]. The NO emissions were exceedingly high for all acquired activated carbons, indicating a high surface reduction potential or a low NO adsorption capacity. The NO_2_ breakthrough curves for nitrogen-amidated activated carbons exhibited prominent deviations from those of unmodified PA carbon, signifying a modification in the NO_2_ adsorption mechanism of this adsorbent. All ammoxidised samples recorded a zero NO_2_ concentration for a specific phase, and only subsequently did the ‘bed breakthrough’ transpire, with a gradual increase in gas concentration. By examining the NO_2_ adsorption curves, it can be observed that when adsorption is performed in the presence of water vapour (Figure 4b), the duration for which the NO_2_ concentration remains constant at zero is increased. This effect confirms the advantageous impact of water vapour on the achieved adsorption capacities. In the PA sample case, the bed breakthrough occurred at the first stage of the adsorption process and the ppm NO_2_ value gradually increased towards the limit value. A prolonged phase was observed during the process where the NO_2_ concentration remained at 0 ppm in the presence of water vapour (Figure 4b), leading to higher adsorption capacities under these conditions (Table 6).

Activated carbon has garnered popularity due to its well-developed specific surface area and the ability to modify its surface by introducing heteroatoms or metals/metal oxides. Numerous researchers have conducted studies on the mechanism of NO_2_ adsorption onto carbon adsorbents [33,34,35,36,37]. As can be observed in the equations below, the process of NO_2_ adsorption depicts a complex sequence of steps where NO molecules are released as a result of NO_2_ reduction.

$$C^{*}+{NO}_{2}\to C\left({NO}_{2}\right)$$

$$C\left({NO}_{2}\right)\to C\left(ONO\right)$$

$$C\left(ONO\right)\to C\left(O\right)+NO$$

It is reasonable to assume that the adsorption of NO_2_ on the studied materials is accompanied by simultaneous processes involving its reduction to NO as well as the adsorption of the produced nitrogen oxide. This phenomenon can be represented by the reactions outlined in our earlier work [38]. Generally, the oxidation reaction of the activated carbon surface results in the generation of carbon oxygen complexes, nitrogen oxides, and nitrogen. The complex C(ONO_2_) may undergo subsequent decomposition, leading to the formation of CO, CO_2_, NO, and NO_2_.

When adsorption is conducted with the inclusion of steam, a blend of nitric acids is generated, enabling the achievement of higher sorption capacities under these circumstances [36]:

$$3{NO}_{2}+H_{2}O\to {2HNO}_{3}+NO$$

$$2{NO}_{2}+H_{2}O\to {HNO}_{3}+{HNO}_{2}$$

The results summarised in Table 1, Table 3 and Table 7 suggest that the determination of the adsorption mechanism of the investigated gas is extremely challenging. We believe that in the case of the activated carbons studied, the adsorption process has both physical and chemical characteristics. For unmodified samples (PA), the high sorption capacities indicate that the adsorption of adsorbate molecules occurs mainly in the pores of the adsorbent. NO_2_ molecules penetrate the structure of the PA sample, suggesting a predominantly physical nature of adsorption. On the other hand, for nitrogen-enriched samples, the interaction between the adsorbate and adsorbent is mainly chemical in nature. This interaction works in two ways. In the case of the NPA, N1PA, PNA, and PN1A samples, a decrease in sorption capacity towards NO_2_ was observed. Conversely, the opposite trend was observed for the PAN and PAN1 activated carbons. This is probably due to the fact that the PAN and PAN1 samples are characterised by one of the highest specific surface areas and also contain the largest amount of nitrogen in their structure. In this case, both physical and chemical interactions take place.

Numerous prior investigations have highlighted the robust NO_2_ adsorption capabilities of activated carbons, as detailed in Table 8. Ghouma et al. [33] conducted research utilising chemically activated carbons derived from olive stones. The activated carbon activated with 50% orthophosphoric(V) acid demonstrated an NO_2_ adsorption capacity of 64.5 mg/g [33]. In the same study, the modification of H_3_PO_4_-activated carbon with a 1 M NaOH solution resulted in an enhanced sorption capacity of 79.1 mg/g [33]. The outcomes reported by Ghouma et al. [33] exhibit comparability to the findings of this study. Moreover, the activated carbon produced from date stones and activated with carbon(IV) oxide displayed notably higher sorption capacity [34]. The researchers [34] established that the creation of porosity in carbon materials, combined with prolonged activation time, enhances NO_2_ adsorption. The adsorbent with a surface area of 655 m^2^/g achieved the highest sorption capacity of 106.9 mg/g towards the target gas. In a study by Belhachemi et al. [35], the removal of NO_2_ from exhaust gases using commercial and modified NORIT GAC 1240 activated carbon was examined. The results indicated the efficacy of the investigated materials as adsorbents for NO_2_ removal. Evaluating the data presented in Table 7, it can be inferred that the PAN1 sample exhibited a sorption capacity more than 50 mg/g lower than the findings reported in [35]. Conversely, the carbonaceous adsorbent obtained from nitrogen-treated pine sawdust pellets demonstrated an NO_2_ adsorption capacity of 45.3 mg/g [36].

To sum up, the outcomes presented in Table 8 highlight that the sorption capacities attained in this study were comparable, and in some cases even higher, than those obtained for adsorbents derived from waste materials or Polish bituminous coal. It is possible that utilising a different type of activator could potentially result in achieving sorption capacities akin to those reported in the study [35]. Furthermore, the research published in [37,39,40] demonstrates the potential of carbon adsorbents with nitrogen admixture in catalysis and fuel cells. These materials can be used effectively in electrocatalysts on a large scale, providing an alternative to currently used materials in industry and promoting a sustainable approach to chemistry.

## 3. Materials and Methods

### 3.1. Materials and Activated Carbon Preparation

The activated carbons were obtained from orthocoking coal sourced from the Jas-Mos mine in Jastrzębie-Zdrój, Poland (Precursor). The adsorbent was sieved through a 0.25 mm mesh sieve and then demineralised (Precursor_dem_). The orthocoking coal demineralisation process was carried out using the Radmacher and Mohrhauer method. During the demineralisation process, 1 kg of the initial material was treated with 2 dm^3^ of hydrochloric acid and left to stand for 24 h. Following this period, the carbon was extensively washed with distilled water until achieving a neutral reaction. Subsequently, it was treated with 2 dm^3^ of hydrofluoric acid and left for another 24 h. After this step, the material was washed again with distilled water to eliminate fluoride ions. The resulting demineralised carbon was then dried at 110 °C until reaching a constant weight. The demineralised carbon was subjected to pyrolysis (P) in a horizontal tube reactor and then heated using a resistance furnace at 500 °C in a nitrogen atmosphere (flow rate 0.170 L/min) for 60 min. The pyrolysis product was activated with sodium hydroxide. The ratio of activator to precursor was 4:1. The chemical activation process was also conducted in a horizontal tube reactor, heated by means of a resistance furnace to 800 °C. Activation (A) was carried out for a duration of 45 min under a nitrogen atmosphere (with a flow rate of 0.330 L/min). The carbon produced was washed in a 5% HCl solution to eliminate any surplus NaOH. Subsequently, it was deposited into the HCl solution and brought to a boil with a reflux condenser, followed by draining and rinsing with hot distilled water until neutral. Finally, the carbon was dried at a temperature of 110 °C.

The nitrogen (N) was introduced during the demineralised precursor stage, pyrolysis product, and activation process under oxidative conditions using a horizontal glass reactor in a resistance furnace. The process occurred at temperatures of 300 (N) and 350 (N1) °C for 4 h. The ammonia flow rate was maintained at a constant of 0.250 L/min, while the air flow rate remained at 0.750 L/min.

### 3.2. Adsorbents Characteristic

A Thermo Scientific FLASH 2000 Elemental Analyser was used to analyse the elemental composition (C, N, H, and S) of all carbon materials. The elemental oxygen content was calculated by difference. This research focused on the catalytic combustion of materials conducted at a temperature of 1200 °C, with exhausted gases detected based on the variance in thermal conductivity. The ash content was evaluated by subjecting the samples to combustion in a microwave muffle furnace (Phoenix model, CEM Corporation, Matthews, IL, USA) at 815 °C for 60 min. All elemental analysis experiments were conducted twice.

The specific surface area of activated carbons was measured using an ASAP 2010 V4.00D instrument. The method used for determining the specific surface area is based on the BET isotherm. Nitrogen was used as the adsorbate for the measurements, with a known specific surface area of ω = 16.2 nm. The measurements were conducted at a temperature of −195.8 °C, and the pressure range (p/p_0_) for the BET equation was set between 0.005 and 0.35. Additionally, measurements were performed to determine the total pore volume and average pore diameter. Total pore volume (V_tot_), micropore volume (V_mic_), mesopore volume (W_mes_), and volume of pores less than 1 nm (V_<1 nm_) were calculated using the density functional theory approach (DFT). The morphology of the activated carbons was performed using a SEM microscope (PHILIPS, The Netherlands).

All of the activated carbons were analysed for basic and acidic functional groups using the Boehm method [32]. The measurements were conducted twice for each adsorbent.

### 3.3. Adsorption Study

Regarding the activated carbons, measurements were conducted to assess the adsorption of iodine and nitrogen(IV) oxide.

The iodine adsorption number was determined following the guidelines of PN-83/C-97,555.04. A portion of activated carbon (0.2000 g) was transferred into a round-bottomed flask. Then, 4 mL of 5% hydrochloric acid solution and 20 mL of iodine solution with a concentration of 0.2 mol/L were added. The flask was securely sealed with a stopper and placed on a mechanical shaker, where the mixture was shaken for 4 min. After this period, the contents of the flask were filtered and rinsed with 50 mL of distilled water to minimise residual iodine on the filter. The resulting solution was titrated with sodium thiosulfate solution until complete discolouration, using starch solution as an indicator. A blank measurement was performed before the actual test. The final result was determined as the arithmetic mean of two parallel determinations. Additionally, for some activated carbons, characteristic parameters for Langmuir and Freundlich models were calculated in accordance with the description provided in the referenced work [41].

The NO_2_ sorption capacity was measured with a QREA PLUS model PGM-2000 gas-monitoring instrument by passing a mixture of NO_2_ and air with 1000 ppm NO_2_ through the bed at a rate of 450 mL/min. The measurements were conducted in dry and wet conditions (70% humidity). A sample with a bed volume of 0.5 mL was inserted into a glass reactor, which was 35 cm long and had an inner diameter of 0.9 cm. A blend of NO_2_ and air was circulated via the bed column, while regulating the flow rate via rotameters. An electrochemical sensor observed the concentration of NO_2_ after passing through the sample bed. The measurement was stopped when the air blend concentration of NO_2_ after passing through the bed reached 20 ppm. To determine the sorption capability in mg NO_2_ per g of adsorbent, the process outlined in [32] was followed. To check the NO_2_ reduction, the concentration of NO was also monitored until 200 ppm (electrochemical sensor limit). As with determining the iodine value, the final result was obtained by calculating the arithmetic mean from two parallel determinations.

## 4. Conclusions

The utilisation of nitrogen-enriched activated carbons and unmodified carbon as selective sorbents for nitrogen(IV) oxide and iodine is investigated in this study. Prior to physicochemical and sorption analyses, the precursor was subjected to demineralisation procedures using concentrated hydrochloric and hydrofluoric acid solutions, leading to the near complete elimination of mineral substances. However, the composition of individual elements was minimally affected by this process.

The successful production of activated carbon with a highly developed specific surface area and distinct microporous surface characteristics is demonstrated in the research. This was achieved through the activation of orthocoking coal sourced from the Jas-Mos mine, using NaOH as the activating agent. An impressive surface area of 2207 m^2^/g and an iodine content of 2045 mg/g were exhibited by this activated carbon. Furthermore, the obtained coal exhibited a notably high elemental carbon content of 96.6 wt. %. A considerably higher presence of acidic surface oxygen groups, in comparison to basic groups, was displayed by the unmodified activated carbon. The coal showed the capability to adsorb 22.8 mg/g of NO_2_ under dry conditions and 69.3 mg/g under wet conditions.

Furthermore, it has been demonstrated by research that the stage at which the nitrogen enrichment process takes place exerts a significant influence on the amount of nitrogen incorporated into carbon. The highest amount of nitrogen is incorporated during the precursor stage, while the least occurs during the activated carbon stage. Moreover, elevating the temperature during the amination process enhances the integration of nitrogen into carbon. Based on the textural parameters of nitrogen-enriched activated carbons, it can be inferred that the samples that underwent ammoxidation during pyrolysis exhibit the lowest specific surface area (1799–1832 m^2^/g), whereas the highest specific surface area is observed for ammoxidised coals in the demineralised precursor stage (2185–2221 m^2^/g). Concerning nitrogen-enriched activated carbons produced through ammonia treatment, the presence of specific functional groups is greatly contingent on the stage at which the coal reacts with the ammonia. The most pronounced advantage of basic groups over acidic ones is displayed by nitrogen-enriched carbons produced through the activation of pyrolysis enrichment with nitrogen. The least substantial advantage is observed in activated carbons enriched with demineralised coal. Adsorption studies have illustrated that nitrogen-enriched carbons obtained via pyrolysis–activation–nitrogen enrichment yield the most favourable adsorption capacities for both NO_2_ and iodine. The highest adsorption capacity for nitrogen oxide(IV) was demonstrated by the sample activated at a temperature of 350 °C through ammoxidation. In dry conditions, this carbon exhibited an adsorption capacity of 23.5 mg/g, while in moist conditions, the capacity rose to 75.0 mg/g. Additionally, the presence of airflow during the NO_2_ adsorption process positively influenced the achieved adsorption capacities, as all adsorbents obtained higher capacities under moist conditions.

## Figures and Tables

**Table 1:** Technical analysis of sample J and JD (%).

Adsorbent	Ash	Moisture	Volatile Compounds
Precursor	2.5	0.9	19.4
Precursor_dem_	0.7	0.0	17.7

**Table 2:** Elemental analysis of all samples (%).

Adsorbent	C^daf^	H^daf^	N^daf^	S^daf^	O^daf^ *	Ash
Precursor	88.4	4.7	1.4	0.3	5.2	2.5
Precursor_dem_	90.6	4.5	1.1	0.2	3.6	0.7
Precursor_demN_	84.2	4.0	6.2	0.2	5.4	0.6
Precursor_demN1_	82.7	3.7	8.3	0.2	5.1	0.6
Precursor_demN_P	85.7	3.7	6.0	0.2	4.4	0.8
Precursor_demN1_P	83.3	3.5	8.1	0.2	4.9	0.9
NPA	95.2	0.5	0.2	0.0	4.1	1.0
N1PA	96.8	0.3	0.1	0.0	2.8	1.1
P	91.0	3.5	0.4	0.3	4.8	0.9
PN	88.2	3.2	2.5	0.3	5.8	0.7
PN1	85.9	3.0	3.6	0.3	7.2	0.7
PNA	94.4	0.9	0.5	0.0	4.2	0.9
PN1A	93.2	0.7	0.4	0.0	5.7	0.8
PA	96.6	0.5	0.1	0.0	2.8	0.8
PAN	94.9	0.4	1.3	0.0	3.4	1.1
PAN1	94.5	0.3	1.4	0.0	3.8	1.0

* dry—dry ash-free basis, *—by difference.

**Table 3:** Textural parameters of the activated carbons.

Adsorbent	Surface Area (m^2^/g)	Micropore Area (m^2^/g)	V_tot_ (cm^3^/g)	V_mic_ (cm^3^/g)	V_mes_ (cm^3^/g)	V_<1 nm_ (cm^3^/g)	Average Pore Diameter (nm)
PA	2207	2199	1.18	1.14	0.13	0.009	2.19
NPA	2221	2177	1.21	1.17	0.18	0.007	2.27
N1PA	2185	2143	1.22	1.16	0.16	0.003	2.28
PNA	1832	1800	1.11	1.01	0.18	0.004	2.35
PN1A	1799	1766	1.07	0.99	0.12	0.005	2.31
PAN	2155	2132	1.14	1.07	0.15	0.003	2.10
PAN1	2153	2128	1.12	1.08	0.17	0.001	2.09

**Table 4:** Acid-base properties of the activated carbons.

Adsorbent	Total Content of Groups (mmol/g)	Acidic Groups (mmol/g)	Basic Groups (mmol/g)
PA	1.74	1.03	0.71
NPA	1.45	0.66	0.79
N1PA	1.38	0.57	0.81
PNA	1.32	0.54	0.78
PN1A	1.33	0.51	0.82
PAN	1.58	0.59	0.99
PAN1	1.66	0.61	1.05

**Table 5:** Iodine number of the carbon adsorbents prepared by chemical activation.

Adsorbent/Material	Active Agent	Iodine Number (mg/g)	References
PA	NaOH	2045	This study
NPA		1699	
N1PA		1721	
PNA		1924	
PN1A		1934	
PAN		2026	
PAN1		2029	
Rice husk	H_3_PO_4_	660.4	[24]
Sawdust	H_3_PO_4_	1628.95	[25]
Noug stalk	H_3_PO_4_	576	[26]
Immature G. hirsutum seeds	H_2_SO_4_	510	[27]
Peltophorum pterocarpum Leaves	H_3_PO_4_ + H_2_SO_4_	1508.76	[28]
Baobab seed hulls	KOH	1854.2	[29]
Bituminous coal	KOH	1736–2095	[30]
Palm kernel shells	NaOH	1062.8	[31]
Pine cones	NaOH	913	[32]

**Table 6:** Langmuir and Freundlich parameters of the adsorption isotherms of iodine adsorption onto activated carbons.

Sample	Langmuir			Freundlich		
	R^2^	q_max_ [mg/g]	K_L_ [L/mg]	R^2^	K_F_ [mg/g(L/mg)^1/n^]	1/n
PA	0.989	2060	0.0011	0.936	1154.52	0.238
NPA	0.990	1941	0.0001	0.974	1040.88	0.285
PNA	0.991	2000	0.0004	0.946	981.97	0.320
PAN	0.999	2000	0.0500	0.903	1872.08	0.052

K_L_—Langmuir adsorption equilibrium constant; q_max_—maximum adsorption capacity of the adsorbent [mg/g], K_F_—Freundlich equilibrium constant; 1/n—intensity of adsorption constant.

**Table 7:** NO_2_ breakthrough capacities of the activated carbons.

Adsorbent	Dry Conditions		Wet Conditions	
	[mg/g]	[mg/cm^3^]	[mg/g]	[mg/cm^3^]
PA	22.8	6.9	69.3	24.8
NPA	10.9	1.9	16.9	3.9
N1PA	10.3	1.7	17.4	3.7
PNA	11.9	2.3	28.0	6.9
PN1A	10.8	2.1	29.8	6.8
PAN	23.0	6.8	73.3	27.4
PAN1	23.5	6.7	75.0	27.5

**Table 8:** Capacity for NO_2_ adsorption on carbon materials in this study and in the literature.

Adsorbent/Material	Active Agent	NO_2_ [mg/g]	References
PA	NaOH	69.3	This study
NPA		16.9	
N1PA		17.4	
PNA		28.0	
PN1A		29.8	
PAN		73.3	
PAN1		75.0	
activated carbon (olive stones)	H_3_PO_4_	64.5	[33]
activated carbon (olive stones)	NaOH	79.1	[33]
activated carbon (date stones)	CO_2_	106.9	[34]
NORIT GAC 1240	steam	127	[35]
modified GAC	(NH_4_)_2_S_2_O_8_, N_2_	136	[36]
carbonaceous adsorbent (pine sawdust pellets)	N_2_	45.3	[37]

## Data Availability

Data are contained within the article.
